# PSMA4 as a Druggable Target in Hidradenitis Suppurativa: Evidence From Mendelian Randomization and Single‐Cell Transcriptomics

**DOI:** 10.1155/mi/4954996

**Published:** 2026-02-10

**Authors:** Siqing Guo, Li Gao, Yanting Sun, Lixin Yin, Peihong Li, Boyun Sun, Jingen Lu

**Affiliations:** ^1^ Department of Proctology, Longhua Hospital, Shanghai University of Traditional Chinese Medicine, Shanghai, China, shutcm.edu.cn; ^2^ Shanghai Fengxian District Traditional Chinese Medicine Hospital, Shanghai, China; ^3^ Department of Gastroenterology, Longhua Hospital, Shanghai University of Traditional Chinese Medicine, Shanghai, China, shutcm.edu.cn

**Keywords:** hidradenitis suppurativa, inflammation, Mendelian randomization, therapeutic targets

## Abstract

Hidradenitis suppurativa (HS) is a chronic inflammatory skin disease with limited treatment options and frequent drug resistance. Novel therapeutic targets are urgently needed. We performed a druggable genome‐wide Mendelian randomization (MR) analysis using blood cis‐expression quantitative trait locus (eQTL) and HS genome‐wide association study (GWAS) data. Colocalization, transcriptomic validation, single‐cell RNA sequencing, and cell–cell communication analyses were integrated to explore gene function and cell‐type specificity. We identified eight genes that showed significant associations with HS through MR analysis. Colocalization analysis further prioritized PSMA4 and MAST3 as the most promising druggable targets for HS. Specifically, PSMA4 (single nucleotide polymorphisms [SNPs] = 10; inverse‐variance weighted [IVW] OR = 1.912, 95% CI: 1.492–2.450, *p* < 0.001; PP.H4 = 0.975) and MAST3 (SNPs = 15; IVW OR = 0.557, 95% CI: 0.453–0.686, *p* < 0.001; PP.H4 = 0.832) exhibited strong statistical associations. Transcriptomic validation revealed that PSMA4 was upregulated and MAST3 was downregulated in HS lesions. Further single‐cell analysis revealed that PSMA4 was predominantly enriched in CD4^+^ T cells and involved in pro‐inflammatory signaling, particularly the tumor necrosis factor (TNF) pathway. PSMA4 and MAST3 are potential therapeutic targets for HS. PSMA4 may promote inflammation via CD4^+^ T cell‐mediated signaling, offering a novel avenue for treatment development.

## 1. Introduction

Hidradenitis suppurativa (HS) is a chronic inflammatory skin disorder characterized by recurrent deep‐seated nodules, abscesses, fistulae, sinus tracts, and scarring, primarily affecting the axillary, inguinal, submammary, and perianal regions. Despite its substantial impact on patients’ quality of life, HS remains underdiagnosed and undertreated. Its prevalence is not well established, with estimates ranging from 0.00033% to 4.1% [1–3]. However, the treatment of HS remains challenging. Pharmacological management typically begins with empirically prescribed antibiotics, yet high‐quality evidence supporting this approach is lacking. Although novel monoclonal antibody therapies have been introduced for HS treatment [4, 5], challenges with therapeutic efficacy remain a significant concern, contributing to poor disease prognosis and reduced quality of life. Consequently, identifying new therapeutic targets to overcome these limitations has become imperative. Addressing this unmet need is crucial for developing innovative treatments capable of enhancing therapeutic outcomes and improving patient quality of life.

The conventional drug development process is both time‐consuming and costly. However, breakthroughs in genomics have revolutionized drug target discovery and validation. Against this backdrop, the concept of the druggable genome was introduced to enable the systematic identification of therapeutic targets with potential for pharmacological intervention. Druggable genes are generally defined as those encoding proteins that can be modulated by pharmacological means; these proteins are either targets of approved drugs or agents currently in clinical development, or they belong to classical druggable protein families such as kinases, receptors, and enzymes [6]. This conceptual framework provides a critical theoretical basis for prioritizing candidate targets with translational potential at the genome‐wide level. Building on this foundation, Mendelian randomization (MR) leverages single nucleotide polymorphisms (SNPs), which represent common genetic variants, as instrumental variables to emulate the design of randomized controlled trials (RCTs) without the need for pharmacological intervention. This approach is founded on the biological principle of genotype‐based randomization, whereby the natural randomization of genetic variants mimics the random allocation in RCTs, thereby providing robust evidence for drug target validation [6, 7]. In particular, in studies on HS, the key role of genetic susceptibility in disease onset highlights the distinct advantages of MR methods. Moreover, this study will integrate single‐cell RNA sequencing (scRNA‐seq) and RNA sequencing technologies to explore the mechanisms linking drug target genes to HS systematically. scRNA‐seq, by analyzing gene expression profiles at the single‐cell level, overcomes the limitations of traditional bulk sequencing in capturing cellular heterogeneity, providing a high‐precision perspective for investigating disease mechanisms [8]. Leveraging this technological advantage, the present study further integrates expression quantitative trait locus (eQTL) analysis to precisely identify critical loci regulating gene expression within specific cell subpopulations by correlating genetic variations with single‐cell gene expression data [9]. This multi‐omics integration strategy provides insight into the cell type‐specific gene regulatory networks involved in the pathogenesis of HS, offering novel opportunities for targeted therapeutic development.

Thus, we conducted a comprehensive druggable genome‐wide MR study to explore potential therapeutic targets for HS. Initially, we performed a two‐sample MR analysis by integrating druggable gene eQTL data from blood with genome‐wide association study (GWAS) data for HS, aiming to identify genes strongly associated with HS. We then employed single‐cell expression analysis to investigate the enrichment of these genes in specific cell types within HS tissue. Finally, we utilized cell communication analysis to explore the potential pathogenic mechanisms of key genes. Our goal is to enhance personalized treatment strategies for HS patients and provide a robust foundation for the development of targeted therapies capable of altering patient outcomes.

## 2. Methods and Materials

### 2.1. Two‐Sample MR of Druggable cis‐eQTL With HS

**Figure 1:**
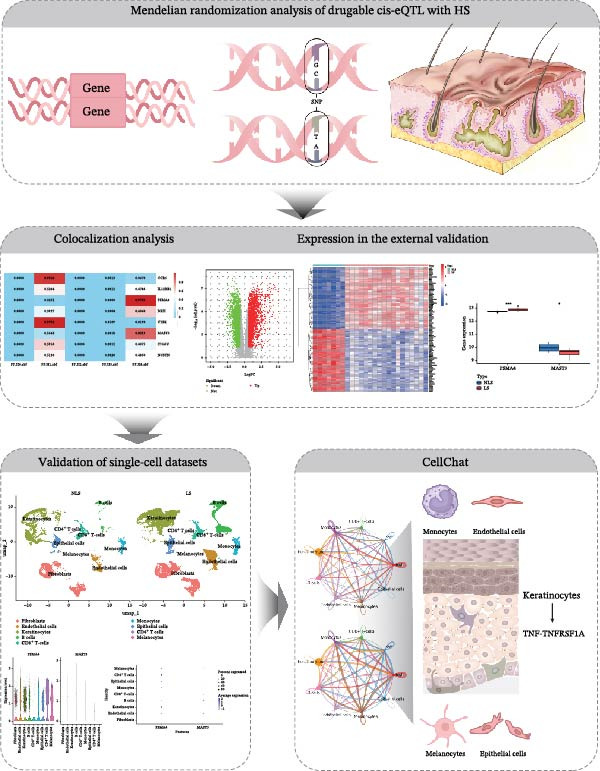
Overview of the study design.

A two‐sample MR analysis was conducted using cis‐eQTL data to proxy genetically predicted expression levels of druggable genes in blood as the exposure, and GWAS data for HS as the outcome, thereby assessing the causal relationship between gene expression and both susceptibility to and progression of HS (Figure 1). Druggable genes are defined as genes encoding proteins with potential for pharmacological modulation, whose sequences or structures exhibit a certain degree of similarity to known drug targets. According to the study by Finan et al. [6] a total of 4479 druggable genes were identified, including 1427 genes encoding targets of approved drugs or agents in clinical development; 682 genes encoding proteins capable of binding known drug molecules or exhibiting structural similarity to approved drug targets; and 2370 genes belonging to key druggable gene families or encoding proteins with more distant similarity to approved drug targets. This diverse repertoire of druggable genes provides a broad pool of candidates for the genetic screening of disease‐relevant therapeutic targets. Subsequently, blood cis‐eQTL data were obtained for 2525 of the 4479 druggable genes from the eQTLGen consortium [10, 11]. The consortium integrates eQTL data from 37 independent cohorts, encompassing 31,684 participants, with the study population predominantly of European ancestry. This eQTL resource was utilized to identify genetic variants associated with gene expression levels in blood; these variants are located within approximately ±1 Mb upstream or downstream of the target genes, and all variants included in the analysis have a minor allele frequency greater than 0.01. The MR analysis was conducted using the TwoSampleMR package [12], ensuring methodological rigor and comparability of our results. The HS outcome data were obtained from the FinnGen database: https://storage.googleapis.com/finngen-public-data-r12/summary_stats/release/finngen_R12_L12_HIDRADENITISSUP.gz, which includes ncase = 1364 and ncontrol = 476,404. Suitable SNPs were selected as instrumental variables (IV) based on strict inclusion and exclusion criteria. We chose strong instrument variants with F‐statistics ≥ 10 and variants with low linkage disequilibrium (LD, *r* ^2^ < 0.1), excluding those that did not meet the threshold of *p* < 5 × 10^−8^. SNPs with incompatible allele codings between the exposure and the outcome (e.g., A/G vs., A/C) were excluded. For palindromic SNPs, forward‐strand alleles were inferred using allele frequency information; in the absence of allele frequency data, palindromic SNPs were directly excluded from the analysis. MR analysis was conducted using the inverse‐variance weighted (IVW), weighted median, and MR‐Egger methods. The Wald ratio estimate was calculated for each SNP. The IVW method provides the maximum statistical power under the assumption that all instruments are valid [13]. In cases where the results from the three MR methods were inconsistent, IVW was used as the primary analysis [14], and the significance threshold was adjusted using false discovery rate (FDR) correction. A series of sensitivity analyses were performed to assess the quality of the MR analysis, including tests for heterogeneity and pleiotropy. Cochran’s Q and MR‐Egger intercept tests were used to evaluate the horizontal pleiotropy and heterogeneity of genes with two or more instruments [15].

### 2.2. Colocalization Analysis Between Druggable cis‐eQTL and HS

We performed a colocalization analysis between the positive results from the MR analysis after FDR correction and HS to assess whether cis‐eQTLs and HS share the same pathogenic variants [16]. Colocalization analysis was conducted using the “coloc” package on genes with positive MR results. Bayesian methods were employed to determine whether the association between gene expression and HS is attributable to common causal variants [17]. The first hypothesis (H0) assumes that there is no significant correlation between SNPs in the cis‐eQTL and HS regions; the second hypothesis (H1) suggests that only cis‐eQTL loci are significantly associated; the third hypothesis (H3) proposes that only HS loci are significantly associated; the fourth hypothesis (H4) posits that SNPs in both the cis‐eQTL and HS regions are significantly associated but driven by distinct causal variants; and the fifth hypothesis (H5) asserts that SNPs in both regions are significantly associated and driven by the same causal variant. For trait‐specific relationships, the prior probability was set to 1 × 10^−4^, and for shared variants, the prior probability was set to 1 × 10^−5^. A posterior probability (PPH4) ≥ 0.8 was considered strong colocalization, and genes with strong colocalization were regarded as potential drug target candidates.

### 2.3. Transcriptomic Profiling Validates Druggable Gene Expression in HS

Transcriptomic dataset GSE72702 includes 17 samples from HS lesions and 13 from nonlesional HS skin. Similarly, the GSE148027 dataset comprises 18 HS lesion samples and 7 nonlesional samples. These external validation datasets were used to verify the expression of key genes.

### 2.4. Validation and Expression in Single‐Cell Datasets

We selected the single‐cell dataset GSE158955 to validate the expression distribution of key genes, which included four HS samples from the axillary and inguinal regions, along with three autologous control samples from perilesional axillary and inguinal skin. First, we performed quality control, setting a minimum of three cells per sample and requiring each cell to express at least 100 genes. Cells with mitochondrial content below 15% were retained to exclude low‐quality cells. Next, we normalized the data using the “Seurat” package’s *LogNormalize* function to mitigate sequencing depth effects and balance expression differences. The 1500 most variable genes were selected for downstream analyses. Subsequently, we applied principal component analysis (PCA). The data were first standardized using the *ScaleData* function, and then the top 20 principal components were computed using the *RunPCA* function. After batch effect removal, these components were retained for further analyses. For clustering analysis, we calculated the adjacency distance between cells using the *FindNeighbors* function and performed clustering using the *FindClusters* function, adjusting the resolution to determine the optimal number of clusters. To visualize the clustering results, we applied uniform manifold approximation and projection (UMAP) via the *RunUMAP* function and plotted the UMAP results using *DimPlot*. Finally, differentially expressed genes (DEGs) for each cluster were identified using the *FindAllMarkers* function, with a minimum expression ratio threshold of 0.25 and a log fold change (logFC) threshold of 1. Additionally, we further analyzed differences in cell types across various groups.

### 2.5. Mechanistic Analysis of CD4^+^ T Cell Subpopulation Regulation by Key Genes

CD4^+^ T cells were classified into high‐expression and low‐expression groups based on whether the key gene was overexpressed. Cell–cell communication analysis was performed to identify potential differences in intercellular communication, upstream and downstream ligand‐receptor interactions, and signaling pathways between the two groups, aiming to elucidate the potential pathogenic mechanisms of the key gene. Using *CellChatDB.human* database, ligand and receptor information was imported, and *subsetDB* was applied to filter database content related to secreted signaling. The categories of ligand‐receptor pairs were visualized using *showDatabaseCategory*. To ensure data quality, the dataset was subset using *subsetData*, and overexpressed genes and interactions within each cell group were identified with *identifyOverExpressedGenes* and *identifyOverExpressedInteractions*. Following data preprocessing, intercellular interactions were calculated, and communication pairs involving fewer than 10 cells were excluded using *filterCommunication*. Pathway‐level communication was inferred through *computeCommunProbPathway*. The results were summarized and visualized using *netVisual_Circle* to illustrate the quantity and intensity of intercellular interactions. We specifically extracted the communication network of each cell population and visualized the communication patterns within individual cell populations. Additionally, a bubble chart was constructed to further illustrate intercellular signaling dynamics.

## 3. Results

### 3.1. MR Results of Druggable Genes and HS

**Figure 2:**
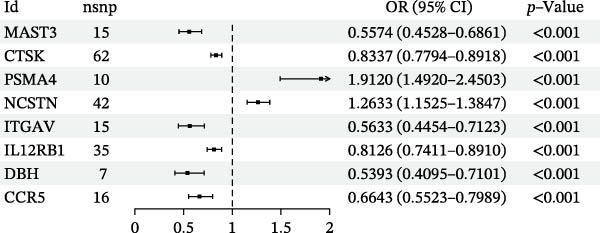
MR results between druggable eQTLs and HS.

We summarized all MR results corrected using FDR and identified 10 genes with FDR‐adjusted *p* < 0.05. Subsequently, we excluded results exhibiting heterogeneity or pleiotropy. Specifically, C4B was excluded based on the heterogeneity test, and CD244 was excluded based on the pleiotropy test. Ultimately, we identified eight druggable genes associated with HS that met the MR criteria (Figure 2). For details, the MR results of druggable genes associated with HS are presented in Supporting Information 1: Table S1, with additional information, including heterogeneity and pleiotropy test results, provided in Supporting Information 2: Table S1.

### 3.2. Results of Colocalization Analysis

**Figure 3:**
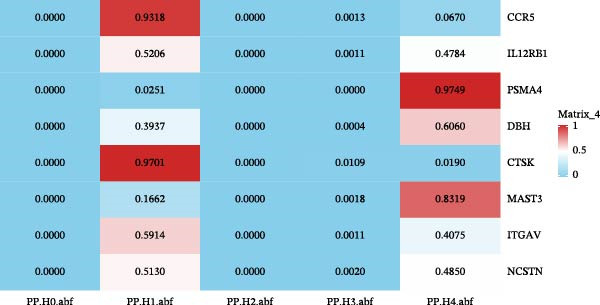
Colocalization plots for nine MR results were generated, each illustrating the significance levels of the five hypotheses with varying probabilities.

For druggable genes with significant MR results, we performed colocalization analysis to calculate the probability of shared causal variants between cis‐eQTLs and HS outcomes. The results of the colocalization analysis indicated that HS susceptibility and the genes PSMA4 and MAST3 might share a causal variant, with the posterior probabilities (PP) satisfying hypothesis 5 (H4 > 0.80) (PSMA4: 97.5%; MAST3: 83.2%). Consequently, based on the MR and colocalization analyses, PSMA4 was identified as a potential drug target associated with an increased risk of HS, while MAST3 was identified as a potential drug target associated with a reduced risk of HS (Figure 3).

### 3.3. Differential Expression Analysis of the Transcriptome

We validated the expression of PSMA4 and MAST3 using transcriptomic datasets GSE72702 and GSE148027. The HS lesion area was designated as the lesion group (LS), and the surrounding healthy skin served as the control group (NLS). The results revealed a significant increase in PSMA4 expression in the LS compared to the NLS and a significant decrease in MAST3 expression in the LS compared to the NLS (Figure 4A,B). Volcano and heat maps for differential expression analysis of the transcriptome are detailed in the Supporting Information (Supporting Information 2: Figure S1).

Figure 4(A) Expression levels of PSMA4 and MAST3 in the bulk transcriptomic dataset GSE72702. Red indicates the LS group, and blue indicates the NLS group. (B) Expression levels of PSMA4 and MAST3 in the bulk transcriptomic dataset GSE148027. Red indicates the LS group, and blue indicates the NLS group. For panels A and B, asterisks denote statistical significance: *p* < 0.05 ( ^∗^), *p*  < 0.01 ( ^∗∗^), and *p* < 0.001 ( ^∗∗∗^). (C) Single‐cell clustering map illustrating the distribution of distinct cell clusters across different cell populations. The NLS group represents healthy skin adjacent to HS lesions, whereas the LS group corresponds to lesional HS skin. (D) Violin plot showing the distribution and expression levels of the core gene across individual cell clusters. (E) Bubble plot visualizing the expression of the core gene within each cell cluster, where bubble size reflects expression intensity and bubble color represents relative expression level.

**Figure:**
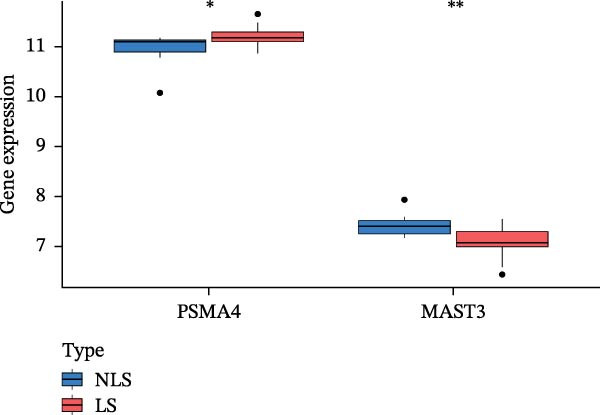
(A)

**Figure:**
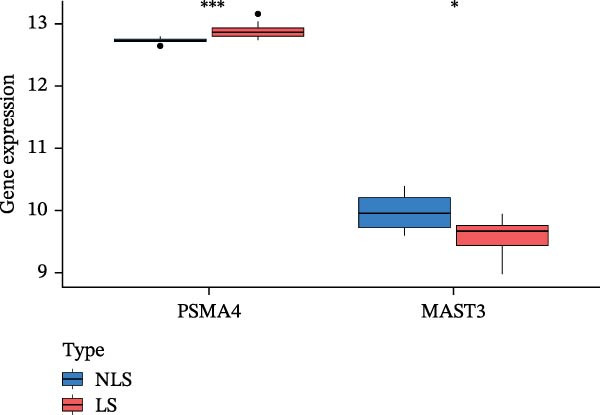
(B)

**Figure:**
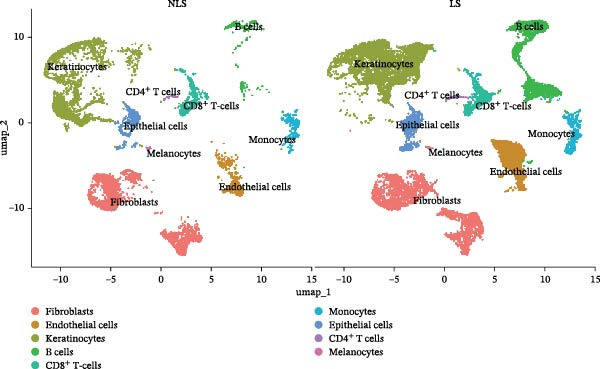
(C)

**Figure:**
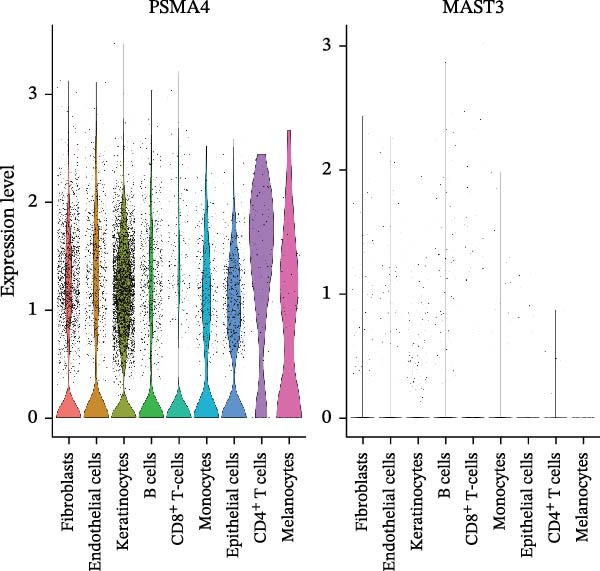
(D)

**Figure:**
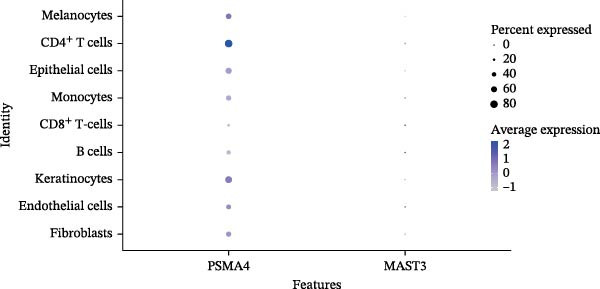
(E)

### 3.4. Validation and Expression Analysis of Single‐Cell Datasets

To further explore the expression pattern of the key risk gene PSMA4 in specific cell types in patients with HS, we utilized scRNA‐seq data from the GSE158955 database. Cells were clustered into different populations based on the expression of marker genes, and the clustering results were visualized using UMAP. A total of nine cell subpopulations were identified, including fibroblasts, endothelial cells, keratinocytes, B cells, CD8^+^ T cells, monocytes, epithelial cells, CD4^+^ T cells, and melanocytes (Figure 4C,D). We observed that PSMA4 was distributed across various cell subpopulations, particularly in CD4^+^ T cells, where it exhibited significant intergroup differences, showing a marked increase in expression (log2FC = 1.178, *p*  < 0.001) (Figure 4E). In contrast, MAST3 did not exhibit significant expression across the examined cell subpopulations.

### 3.5. Differential Cellular Communication Between PSMA4‐High and PSMA4‐Low Expressing CD4^+^ T Cells

CD4^+^ T cells were categorized into PSMA4‐high and PSMA4‐low expression groups to examine differences in cellular communication between these two populations. Our analysis revealed that cells in the PSMA4‐high expression group exhibited significantly greater interaction levels compared to the PSMA4‐low expression group, particularly with keratinocytes, monocytes, endothelial cells, and epithelial cells. Furthermore, the PSMA4‐high expression group showed enrichment in the tumor necrosis factor (TNF) pathway, IL16 pathway, PARs pathway, and CD40 pathway compared to the PSMA4‐low expression group. Specifically, the TNF pathway was found to span multiple downstream receptors, with the primary ligand–receptor pairs being TNF‐TNFRSF1A and TNF‐TNFRSF1B. Based on expression levels (Figure 5A,B), the number and intensity of interactions between the high/low expression groups and other cell types are detailed in Figure 5C,D. Differences in cellular communication between the two expression groups are illustrated in a bubble plot (Figure 5E), and the ligand–receptor relationships and upstream and downstream regulatory networks of the TNF pathway are presented in Figure 5F,G,H.

Figure 5Single‐cell profiles of PSMA4‐high and PSMA4‐low expression groups. (A) UMAP plot showing the classification based on PSMA4 expression. Different colors indicate distinct groups and cell types, with “high” representing the PSMA4‐high expression group and “low” representing the PSMA4‐low expression group. (B) UMAP topographic map classified by PSMA4 expression. Different colors represent various groups and cell types, with “high” denoting the PSMA4‐high expression group and “low” indicating the PSMA4‐low expression group. Clustered regions on the map highlight areas with the most significant differences. (C) Graph illustrating the number of cell–cell interactions between the PSMA4‐high and PSMA4‐low expression groups. (D) Graph depicting the interaction intensity between cells in the PSMA4‐high and PSMA4‐low expression groups. (E) Bubble plot visualizing the cellular communication differences between the PSMA4‐high and PSMA4‐low expression groups. (F) Heatmap of signaling senders, receivers, and regulators between groups, where the PSMA4‐high expression group serves as the primary signal sender. (G) Contribution intensity diagram of upstream and downstream ligand–receptor interactions. (H) Diagram showing PSMA4‐high expression group cells acting as signal senders, influencing downstream receptors through the TNF signaling pathway.

**Figure:**
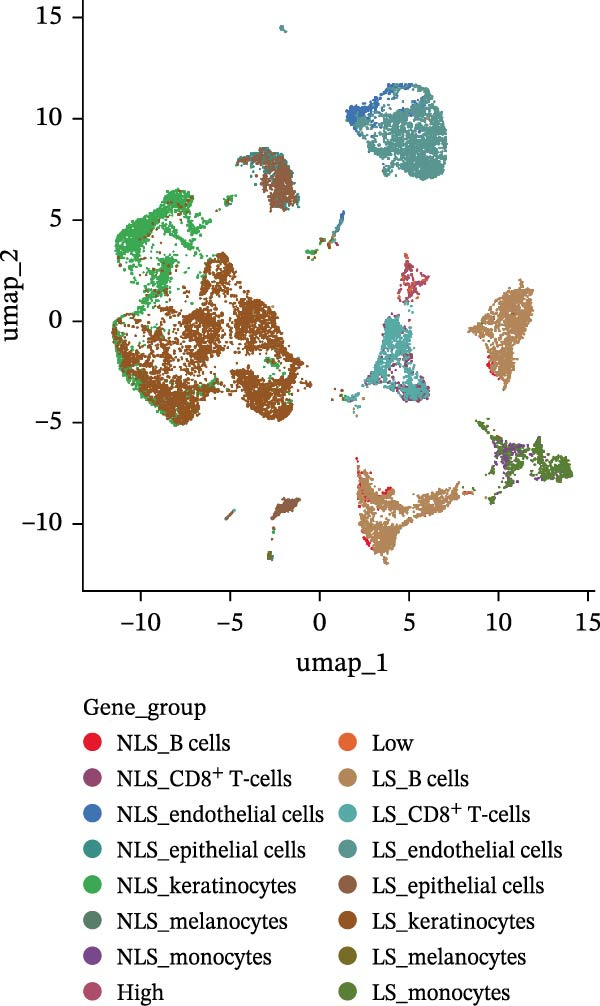
(A)

**Figure:**
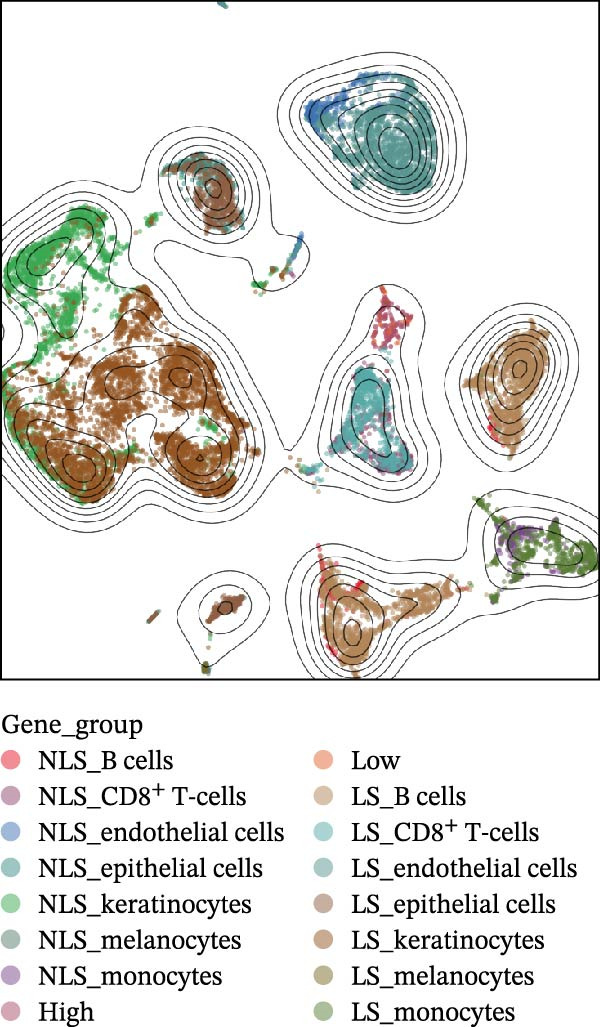
(B)

**Figure:**
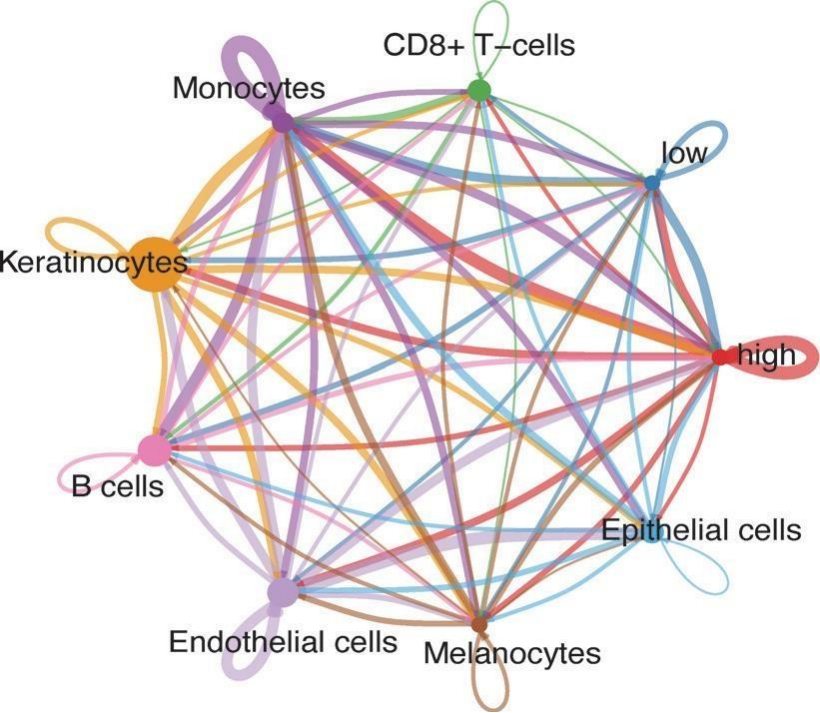
(C)

**Figure:**
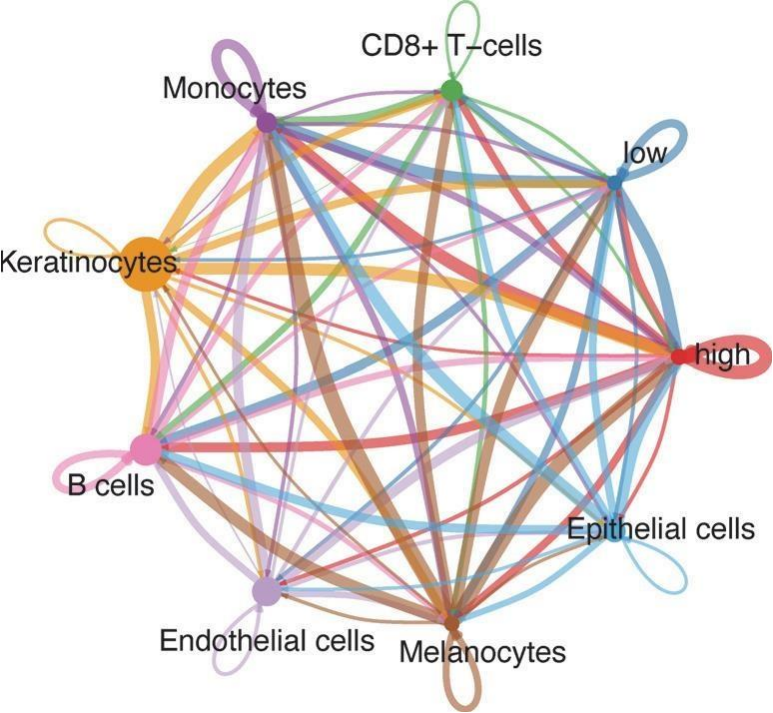
(D)

**Figure:**
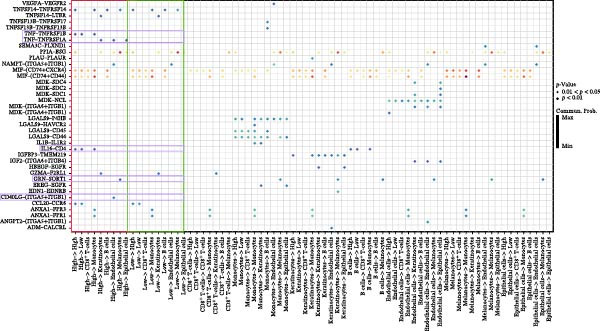
(E)

**Figure:**
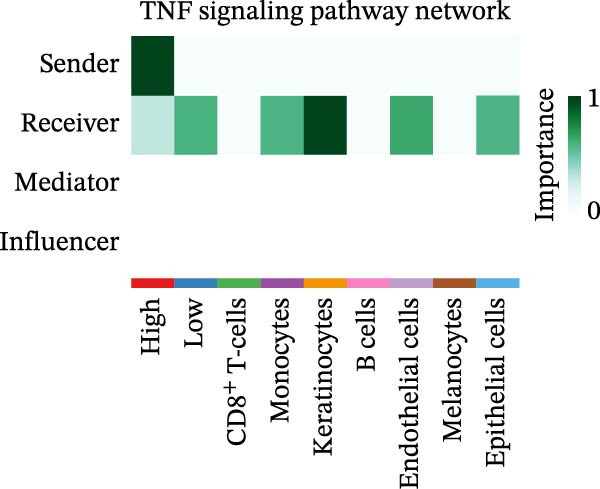
(F)

**Figure:**
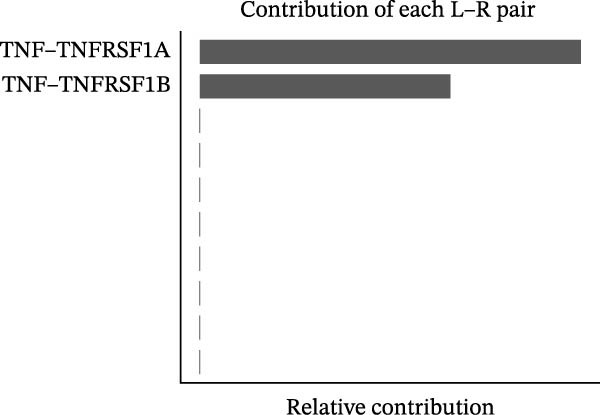
(G)

**Figure:**
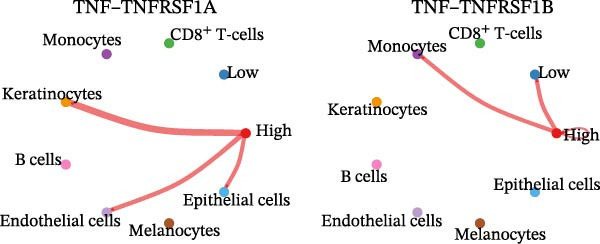
(H)

## 4. Discussion

HS is a chronic inflammatory skin disease characterized by follicular occlusion, recurrent inflammatory nodules, and sinus tract formation, for which approved targeted therapies remain limited. Through large‐scale MR and colocalization analyses, druggable genes were integrated with HS GWAS data, leading to the identification of two potential therapeutic targets, PSMA4 and MAST3, with robust genetic support, thereby providing a rationale for targeted intervention in HS. In the early stages of HS, excessive hyperkeratosis of the terminal hair follicle results in follicular epithelial thickening, with the accumulation of keratinous material giving rise to cystic dilation. Subsequent follicular rupture releases tissue debris and microbial products, which trigger an acute inflammatory response dominated by neutrophils. Macrophage‐derived TNF‐α and interleukin‐1β (IL‐1β) paracrinally activate the nuclear factor kappa‐B (NF‐κB) signaling pathway in keratinocytes, inducing the production of CXCL1 and IL‐8 and ultimately promoting extensive neutrophil infiltration and inflammatory nodule formation [18–20].

PSMA4 is a key member of the proteasome (PSM) protein family. As an essential subunit of the 20S proteasome core particle, the α4 subunit encoded by PSMA4 plays an indispensable role in maintaining proteasome function [21, 22]. Accumulating evidence indicates that this protein regulates protein degradation via the ubiquitin–proteasome system and serves a central role in inflammatory regulation, signal transduction networks, and cellular stress–response mechanisms [23]. Gene knockout studies have demonstrated that downregulation of PSMA4 expression markedly reduces proteasome activity, leading to abnormal intracellular accumulation of ubiquitinated proteins [24, 25]. Such disruption of proteostasis constitutes an important pathological basis for a range of diseases, including cardiomyopathy, heart failure, chronic obstructive pulmonary disease, and sepsis [26–28]. In tumor metabolism, PSMA4 has been implicated in metabolic reprograming through the enhancement of oxidative phosphorylation, thereby facilitating the adaptation of myeloma cells to hypoxic conditions. This process is accompanied by aberrant activation of hypoxia‐inducible factor‐1α (HIF‐1α) signaling, which promotes anti‐apoptotic capacity and contributes to the development of resistance to proteasome inhibitors, such as bortezomib [29]. In skin biology, PSMA4 specifically regulates keratinocyte function [30]. Its downregulation during hypertrophic scar formation is associated with dysregulation of extracellular matrix (ECM)–receptor interaction pathways [31, 32]. Moreover, PSMA4 gene polymorphisms have been linked to scar susceptibility, and MR analyses suggest that PSMA4 may represent a novel target for facial aging [33, 34]. Mechanistic studies further demonstrate that PSMA4 maintains the balance between cell proliferation and apoptosis by coordinating proteasomal degradation of transcription factors, cyclins, and apoptosis‐related proteins [35]. Multiple proteasome inhibitors targeting PSMA4 have been developed, including ixazomib, bortezomib, carfilzomib, oprozomib, marizomib, cotinine, and ixazomib citrate, all of which were designed to inhibit 20S proteasome activity [36]. By disrupting protein degradation, these agents induce the accumulation of toxic proteins and trigger cell death, thereby demonstrating substantial therapeutic efficacy in diseases such as multiple myeloma. Notably, proteasome inhibitors targeting components including PSMA4, exemplified by Bortezomib, exhibit the potential to modulate excessive inflammation and regulate cytokine expression induced by diverse stimuli [37–39]. Marizomib is an irreversible, pan‐proteasome inhibitor that acts on multiple catalytic sites of the 20S proteasome, including the β5, β2, and β1 subunits, thereby suppressing global proteolytic activity. As PSMA4 is an integral component of the 20S proteasome core complex, inhibition of this complex by marizomib indirectly affects proteasome‐mediated regulation of inflammation and cellular homeostasis [40]. Disruption of this protein quality control system may explain its pivotal role in the pathogenesis and progression of HS. Based on these findings, proteasome inhibitors, such as Marizomib, emerge as promising candidate drugs targeting PSMA4, advancing their clinical evaluation in the treatment of HS.

MAST3 (microtubule‐associated serine/threonine kinase 3) is a multifunctional kinase whose kinase domain–mediated phosphorylation activity plays a critical role in inflammation, signal transduction, and immune regulation [41, 42]. Accumulating evidence indicates that MAST3 promotes the progression of inflammatory bowel disease through modulation of the NF‐κB signaling pathway. In inflamed mucosal tissues from patients with ulcerative colitis, MAST3 expression is significantly upregulated and facilitates serine/threonine residue phosphorylation of signaling proteins along the TLR4–NF‐κB axis [43]. In rheumatoid arthritis, overexpression of MAST3 enhances proliferation of fibroblast‐like synoviocytes and inflammatory cytokine release, whereas downregulation of MAST3 by microRNA‐125a‐3p suppresses both the NF‐κB and Wnt/β‐catenin pathways, thereby attenuating disease progression [44, 45]. Furthermore, aberrant methylation of MAST3 (e.g., hypermethylation at the cg02814054 locus) has been associated with an increased risk of obesity [46]. In psoriasis models, twin‐based peripheral blood reduced representation bisulfite sequencing (RRBS) combined with RNA sequencing further corroborates the involvement of MAST3 in cutaneous immune dysregulation [47]. To date, no studies have reported the existence of specific small‐molecule activators that directly target MAST3. Available evidence suggests that certain classical signaling pathway modulators may indirectly influence MAST3‐associated kinase networks through upstream mechanisms. For example, Forskolin and IBMX elevate intracellular cyclic AMP (cAMP) levels and thereby activate protein kinase A (PKA) signaling, whereas A‐769662 is a well‐characterized activator of AMP‐activated protein kinase (AMPK); these pathways play important roles in inflammatory and immune regulation [48–50]. However, direct evidence demonstrating specific interactions between these compounds and MAST3, or their ability to directly modulate MAST3 kinase activity, is currently lacking. Therefore, the potential effects of these agents on MAST3 require further experimental validation.

The present study employed MR to elucidate causal relationships between druggable genes and HS, thereby providing genetic evidence for potential therapeutic targets. However, several limitations should be acknowledged. First, the linear causal assumption underlying MR may not fully capture the biological effects of short‐term, high‐dose interventions. Second, as the analysis was based primarily on GWAS data derived from populations of European ancestry, the generalizability of the findings to other ethnic groups remains to be established. Third, although the use of cis‐eQTL enhanced target specificity, trans‐eQTL effects and epigenetic regulatory networks were not incorporated, potentially leading to the omission of key biological pathways. In addition, owing to the lack of validation using GWAS data for protein quantitative trait loci (pQTLs), the current findings remain confined to the gene expression level, underscoring the need for future studies to extend the investigation from genes to proteins. Moreover, while MR provides causal inference, the pharmacokinetics, off‐target effects, and safety profiles of the proposed targets must be evaluated through RCTs. Therefore, future research should build upon MR‐derived discoveries and transition toward drug development by integrating molecular experiments, animal models, and clinical trials to systematically assess target efficacy and safety, ultimately facilitating the development of more effective and safer therapies for HS.

## 5. Conclusion

This study utilized MR analysis to uncover potential causal relationships between druggable genes and HS, providing an innovative framework for target identification in the era of precision medicine. Nonetheless, further exploration of its clinical value necessitates large‐scale, multi‐ethnic studies, experimental validations, and RCTs to comprehensively evaluate the biological mechanisms, pharmacological properties, and translational potential of the identified targets, thereby providing evidence‐based support for precision medicine.

## Author Contributions

Siqing Guo contributed to conceptualization, methodology, software, writing – original draft. Li Gao contributed to data curation, writing – original draft. Yanting Sun contributed to software development, visualization, and validation. Lixin Yin contributed to the resources, supervision. Peihong Li contributed to software development, visualization, and contributed to writing – review and editing. Boyun Sun contributed to supervision, and writing – review and editing. Jingen Lu contributed to funding acquisition, supervision, and writing—review and editing.

## Funding

This study was funded by Shanghai Municipal Health Commission‐Shanghai Sinus Fistula Disease Research Center (2023ZZ02003); High‐level key discipline of Traditional Chinese Medicine (ZYYZDXK‐2023064).

## Disclosure

All authors reviewed and approved the final manuscript.

## Ethics Statement

The research employed publicly accessible databases, and all incorporated studies received approval from their respective ethics review boards.

## Conflicts of Interest

The authors declare no conflicts of interest.

## Supporting Information

Additional supporting information can be found online in the Supporting Information section.

## Supporting information


**Supporting Information 1** Table 1: MR results of druggable genes associated with HS.

**Supporting Information 2** Table S1: Heterogeneity and pleiotropy test results of MR analysis for druggable genes associated with HS. Figure S1: Transcriptomic Analysis: (A, C) Heatmaps from datasets GSE72702 and GSE148027 display differentially expressed genes (DEGs), with top annotations indicating the NLS group (blue) and the LS group (red). (B, D) Volcano plots from GSE72702 and GSE148027 categorize genes based on their expression patterns: upregulated genes are shown in red, downregulated genes in blue, and with non‐significant differences in expression in gray.

## Data Availability

Publicly available datasets were analyzed in this study. These data can be found here: (GEO, https://www.ncbi.nlm.nih.gov/geo/), (The eQTLGen consortium, https://www.eqtlgen.org/), (The FinnGen database, https://storage.googleapis.com/finngen-public-data-r12/summary_stats/release/finngen_R12_L12_HIDRADENITISSUP.gz,). Details of specific data items are provided in the manuscript. R scripts for analyzing data are available upon reasonable request.
