# Supplementary material for: PSMA4 as a Druggable Target in Hidradenitis Suppurativa: Evidence From Mendelian Randomization and Single‐Cell Transcriptomics
Source: Mediators Inflamm. 2026 Feb 10;2026:4954996. doi: 10.1155/mi/4954996 (PMC12891442; doi:10.1155/mi/4954996)
Supplement: Supplementary file 2 — Supporting Information 2 Table S1: Heterogeneity and pleiotropy test results of MR analysis for druggable genes associated with HS. Figure S1: Transcriptomic Analysis: (A, C) Heatmaps from datasets GSE72702 and GSE148027 display differentially expressed genes (DEGs), with top annotations indicating the NLS group (blue) and the LS group (red). (B, D) Volcano plots from GSE72702 and GSE148027 categorize genes based on their expression patterns: upregulated genes are shown in red, downregulated genes in blue, and with non‐significant differences in expression in gray. [file MI-2026-4954996-s001.docx]

**Supplementary materials**

| Gene Name | MR-PRESSO  p-Value | Heterogeneity | | Pleiotropy | |
| --- | --- | --- | --- | --- | --- |
|  |  | Cochran's Q | p-Value | egger intercept | p-Value |
| MAST3 | 0.503 | 13.839 | 0.462 | -0.033 | 0.258 |
| CTSK | 0.547 | 58.594 | 0.564 | -0.016 | 0.382 |
| PSMA4 | 0.74 | 5.459 | 0.793 | 0.032 | 0.440 |
| NCSTN | 0.432 | 42.468 | 0.408 | 0.030 | 0.099 |
| ITGAV | 0.512 | 14.478 | 0.415 | -0.034 | 0.423 |
| IL12RB1 | 0.833 | 25.949 | 0.837 | -0.006 | 0.700 |
| DBH | 0.682 | 3.996 | 0.677 | -0.041 | 0.560 |
| CCR5 | 0.922 | 8.160 | 0.917 | -0.020 | 0.556 |

**Supplemental Table 1.** Heterogeneity and pleiotropy test results of MR analysis for druggable genes associated with HS.


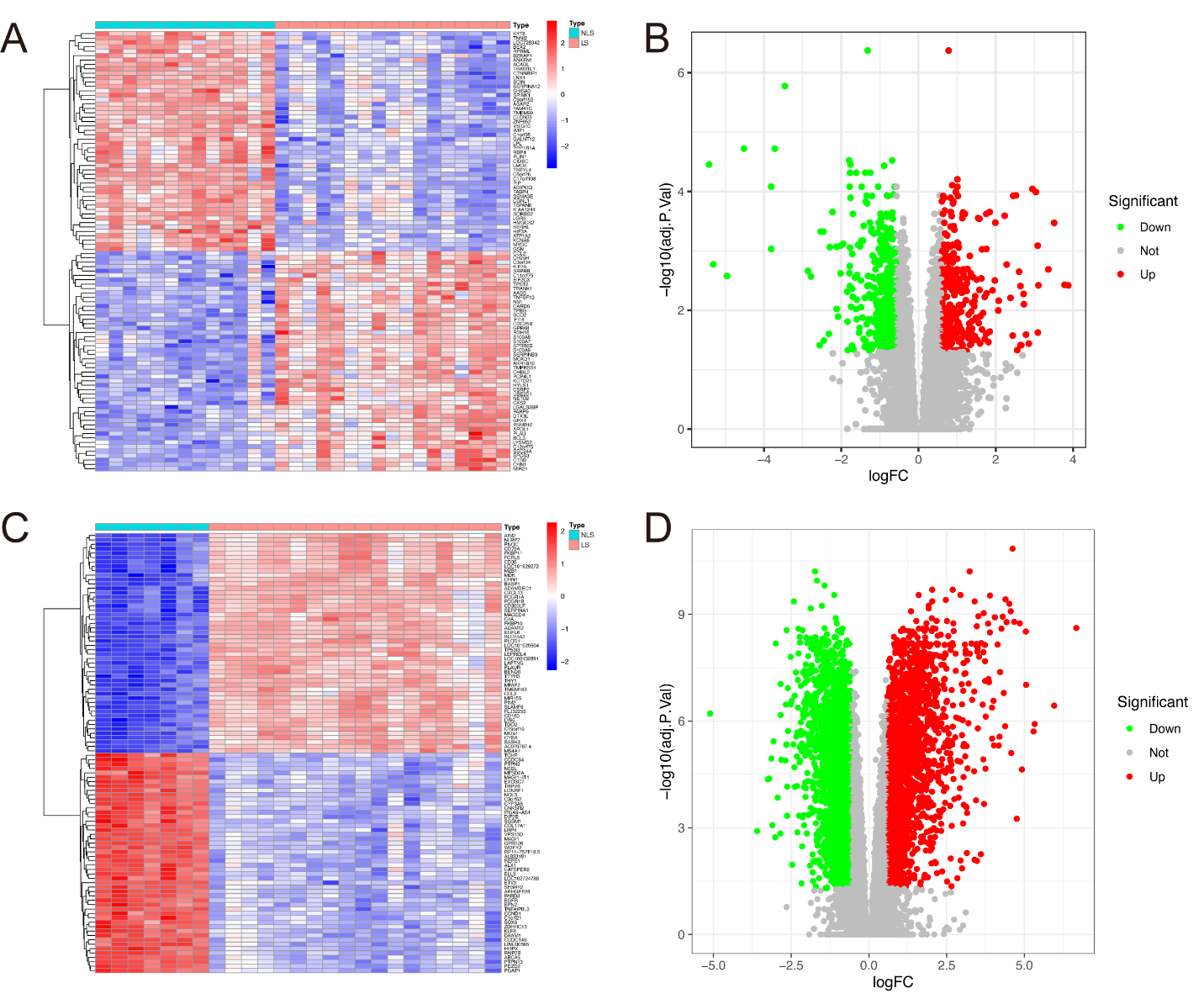


**Supplemental Figure 1**. Transcriptomic Analysis:(A, C) Heatmaps from datasets GSE72702 and GSE148027 display differentially expressed genes (DEGs), with top annotations indicating the NLS group (blue) and the LS group (red). (B, D) Volcano plots from GSE72702 and GSE148027 categorize genes based on their expression patterns: upregulated genes are shown in red, downregulated genes in blue, and with non-significant differences in expression in gray.
